# Mechanisms of impact of web-based support and self-monitoring to augment and maintain physical activity levels: a qualitative study exploring participants’ interactions with the e-coachER, a web-based support programme for people attending exercise referral schemes

**DOI:** 10.1136/bmjopen-2023-080472

**Published:** 2024-10-29

**Authors:** Jeffrey D Lambert, Sarah Gerard Dean, Rohini H Terry, Nigel Charles, Colin Greaves, John L Campbell, Adrian Taylor

**Affiliations:** 1 Department for Health, University of Bath, Bath, UK; 2 Medical School, University of Exeter, Exeter, UK; 3 School of Sport, Exercise and Rehabilitation Sciences, University of Birmingham, Birmingham, UK; 4 Peninsula Medical School, Faculty of Health, University of Plymouth, Plymouth, UK

**Keywords:** qualitative research, chronic disease, public health, behavior, internet

## Abstract

**Abstract:**

**Objectives:**

e-coachER was a web-based intervention designed to support uptake and maintenance of physical activity for people attending exercise referral schemes (ERS) for weight loss, diabetes, hypertension, osteoarthritis or a history of depression/low mood. The aim of this study was to explore the mechanisms of impact of the e-coachER intervention, specifically how participants interacted with e-coachER and the key mediators of increased physical activity.

**Design:**

This was a qualitative one-on-one interview study. Interviews were audio-recorded and transcribed and analysed using thematic analysis.

**Setting:**

UK primary care ERS.

**Participants:**

A purposive sample of adult patients randomised to the intervention arm of the e-coachER randomised controlled trial.

**Results:**

Twenty-six participants (20 female), who had logged on to e-coachER at least once were recruited, resulting in a total of 38 interviews (mean duration 48 min). Four broad, inter-related themes were generated from the data (1) catalyst for change, (2) goals and aspirations, (3) support and (4) engagement with the e-coachER programme. Most participants who took part in e-coachER were already motivated to improve their health and perceived e-coachER as an additional source of motivation and accountability. Many felt that the opportunity to set goals and self-monitor supported participant’s competence and autonomy by enabling them to progress at their own pace. Many participants reported on how e-coachER helped them to foster a sense of relatedness by encouraging them to seek support from others. Finally, e-coachER was regarded as being generally accessible and engaging. Despite this, some found it too simplistic, and others found it hard to maintain engagement over time.

**Conclusions:**

The e-coachER intervention seemed to be generally motivating in the early stages of initiating behaviour change, but engagement waned over time. Our findings highlight how important an online package might be in supporting behaviour change while also highlighting the challenges of achieving sustained physical activity changes.

**Trial registration number:**

ISRCTN15644451.

**STRENGTHS AND LIMITATIONS OF THIS STUDY**

- Participants with a range of health conditions were recruited for the interviews.
- We were unable to conduct a longitudinal analysis; therefore, we combined data from different time points without considering within-person changes over time.
- We were unable to conduct interviews towards the end of the trial to explore participants’ experiences related to their maintenance of physical activity.

## Background

 Musculoskeletal, metabolic and mental health conditions cost UK health services around £1.2 billion annually.^1^ Social prescribing aims to enhance community well-being by linking patients with non-medical sources of support within the community.^2^ Physical activity is one such source of support and has shown to be effective for treating a range of musculoskeletal, metabolic and mental health disorders including depression,^3^ obesity,^4^ type 2 diabetes,^5^ hypertension^6^ and osteoarthritis.^7^ Exercise referral schemes (ERS) are a key subdomain of social prescribing, often prescribed within primary care to support patients with musculoskeletal, metabolic and mental health disorders to increase their physical activity. However, a meta-analysis of eight randomised controlled trials (RCTs) found that ERS only generated small increases in the number of people achieving 150 min of moderate activity per week when compared with usual care,^8^ with most studies involving only relatively short follow-up periods. Many patients also fail to engage with ERS due to cost, inconvenience, the limited time frame of ERS and lack of appeal.^9^

Digitally delivered behaviour change interventions with human support have been shown to support moderate increases in physical activity and may circumvent many of the barriers of ERS.^10 11^ Systematic reviews have also shown digital interventions improve physical activity for people with obesity^12^ and have been shown to be feasible for improving physical activity in low mood/depression.^13 14^ However, the literature is much sparser and more heterogeneous, with a recent review including only nine studies, focussing on a range of different mental health conditions such as schizophrenia, bipolar disorder and depression.^13^

Digital interventions could, therefore, support the uptake of physical activity for patients attending ERS. The e-coachER intervention was a web-based programme on the LifeGuide platform (www.LifeGuideonline.org), which aimed to increase moderate-to-vigorous physical activity (MVPA) among participants attending ERS. Intervention group participants received a package including a user guide, pedometer and fridge magnet for recording MVPA. The user guide facilitated website access and interactive features to boost motivation for ERS and physical activity. The programme featured seven ‘Steps to Health’ sessions, each taking 5–10 min weekly. Achieving step 5 (goal setting and review) was considered a sufficient intervention dose to impact MVPA. e-coachER’s comprehensive approach leveraged multiple behaviour change techniques to enhance engagement and activity levels, regardless of ERS participation. Participants had access to e-coachER throughout the entire 12-month study period.

The e-coachER (exercise referral) intervention was informed by Self-determination Theory (SDT),^15^ which proposes that intrinsic motivation drives engagement in a behaviour. Intrinsic motivation is driven by satisfaction of three core psychological needs, competence, autonomy and relatedness. Evidence-based behaviour change techniques that target these psychological needs were therefore incorporated into e-coachER.^16 17^ For example, e-coachER encouraged participants to consider the benefits of physical activity and deal with ‘setbacks’ (competence), to seek social support to augment and maintain physical activity (relatedness), and to set goals and self-monitor physical activity using the pedometer provided (autonomy).

In a pragmatic multicentre RCT, e-coachER had a small, non-significant effect on device-assessed MVPA (recorded in ≥10 min bouts) at 12 months and no effect on ERS uptake compared with usual ERS.^11 18^ However, secondary analyses revealed that e-coachER reduced depression^19^ and increased device-measured MVPA at 12 months via increased perceived importance, action planning and self-monitoring at 4 months.^20^

The qualitative investigation aimed to explore how e-coachER influenced mechanisms of behaviour change specified in our logic model.^11^ Specifically, how did patients with chronic conditions attending an ERS interact with e-coachER and to what extent did the intended mechanisms of competence, relatedness and autonomy mediate the effect of e-coachER on behaviour change and maintenance of any increases in physical activity.

## Methods

### Design

We conducted a qualitative one-on-one interview study. The reporting follows the Standards for Reporting Qualitative Research framework.^21^ We adopted a pragmatic research paradigm as this was deemed most useful to meet the core objectives to address practical issues and put emphasis on the participant’s experience as a primary means of building knowledge.^22^

### Participants

Participants were recruited from the 144 adult patients referred from primary care to a UK ERS and were taking part in the intervention arm of the e-coachER RCT. The e-coachER RCT was a multicentre, parallel two-group RCT with 1:1 individual allocation to usual ERS alone (control) or usual ERS plus web-based behavioural support (e-coachER). The detailed recruitment pathways for e-coachER are reported elsewhere.^11 18^ We required participants to self-report that they were both physically inactive and identify a primary reason for a referral from the following: the need to lose weight, diabetes, hypertension, osteoarthritis or a history of depression/low mood. The intervention included a welcome pack, user guide, pedometer and a fridge magnet with tear-off strips to allow patients to record daily physical activity. The digital component of e-coachER comprised seven brief ‘steps to health’, each having interactive components, allowing participants to record their physical activity, set and review weekly activity goals and receive feedback. The qualitative study commenced during the internal pilot phase of the RCT and ran in parallel to the main trial during the intervention period and for the initial follow-up period. We purposefully sampled participants randomised to the intervention arm of the e-coachER RCT who had logged on at least once to ensure representation across the three sites (Greater Glasgow, West Midlands and Southwest England, including Plymouth, Cornwall and Mid-Devon), as well as across the five clinical conditions, gender and age. We also recorded participant’s access to the internet and their self-reported confidence in using information technology.

### Patient and public involvement

The e-coachER intervention was developed by researchers at the University of Southampton, Plymouth and Exeter and with patient and public involvement input, using the LifeGuide platform (www.lifeguideonline.org).^23^,^25^

### Procedure

Potential participants were sent an email inviting them to participate in one-to-one interviews. Those expressing interest were then telephoned or emailed by a researcher (NC or RHT) who explained the interview purpose and process; they were then invited to take part in an initial interview as well as to give permission to be contacted for up to three follow-up telephone interviews distributed throughout the intervention period. This serial interview approach was adopted to capture participants’ changing experiences, perceptions and needs of the e-coachER intervention over time.^26^ Semi-structured interviews were conducted over the telephone or in person. Since most interviews were carried out by telephone, an informed consent form for the qualitative interview component was read point-by-point to the participant and signed by the researcher. Participants were also invited to take part in follow-up interviews that were arranged to capture their experiences of moving through the e-coachER steps. The interview topic guides were informed directly by the logic model to capture key information relating to intervention components, delivery, change in motivation/behaviour, contextual influences and outcomes (see online supplemental appendix 1). The topic guides were designed iteratively and reflected different stages of the trial and the intervention. The initial focus was on the recruitment and sign-up process during the internal pilot phase of the RCT. Later, topic guides were designed to capture participants’ experiences of the components of e-coachER as they progressed through the intervention. Questions were asked about participant’s experiences of participating in the RCT, receiving the welcome pack, logging on to e-coachER and their experiences of the pedometer, recording and setting goals and their progression through the intervention steps (see online supplemental appendix 2 for topic guide). Interviews were audio-recorded, transcribed verbatim and anonymised, with any personal data or ways of identifying participants removed; brief field notes were also made at the time of the interview. Transcripts were imported into NVivo V.11 for data management.

### Analysis

Data were thematically analysed both inductively and deductively at a semantic level, drawing on the six phases of thematic analysis.^27 28^ Transcripts were read repeatedly, annotated (NC and RHT) to gain familiarity with the data, and fully coded by RHT using NVivo following an initial period of coding by NC. The inductive analysis included codes from the data based on participants’ experiences, whereas the deductive analysis focused on behavioural theories (eg, SDT) related to initiating and maintaining healthy lifestyle choices. NVivo nodes initially related directly to the interview topic guide questions were then considered and discussed in depth concerning the theoretical underpinnings of e-coachER (RHT and JDL). Additional verification of emerging analyses occurred through discussion (RHT, JDL and SGD) to reach a consensus on themes and data presented to represent these themes.

## Results

We recruited 26 participants who had logged on to e-coachER at least once. Most participants were female, primarily referred for weight loss, with ages ranging from 28 to 72 years (table 1). Overall, 36% of participants did not log in to e-coachER, and 36% engaged enough to complete at least one goal review, requiring over 4 weeks of interaction. The mean number of logins was 14.1 (SD 16.7), and among those who completed a goal review, the mean number of reviews was 14.4 (SD 13.8). The mean time spent on e-coachER was 48.4 (SD 41.9) min for those who registered and 43.3 (SD 37.3) min for those who completed a goal review.^11 18^ Of the 26 recruited, 7 completed more than one interview several weeks apart: 1 participant completed four interviews, 3 completed three interviews and 3 completed two interviews. We carried out 38 interviews, with 11 participants from site 1, 9 from site 2 and 6 from site 3. Three participants completed an interview after completing step 3, six after completing step 4, six after completing step 5 and 11 after completing steps 6 and 7. The telephone interviews lasted between 16 and 80 min, with a mean length of 48 min. Due to the limited number of participants providing multiple interviews, it was not possible to analyse changes in participant experiences over time.

**Table 1:** Participant characteristics (n=26) involved in interviews

Characteristic	Category	
Gender (n)	Female	20
	Male	6
Health condition (n)	Weight loss only	5
	Weight loss plus other morbidities but not low mood	4
	Weight loss and low mood only	7
	Low mood only	2
	Low mood and other morbidity but not weight loss	1
	Weight loss plus low mood and other morbidities	5
	No low mood, not weight loss, other physiological conditions	2
Age range (years)	Female	28–69
	Male	39–72
Participants at each research site (n)	Plymouth	11
	Birmingham	9
	Glasgow	6
Access to IT facilities (n)	Home/Work access/Mobile	22
	Mobile not home access	3
	Public only, not mobile access	1

ITinformation technology

### Themes

Four broad, inter-related themes were discerned from the data, describing how participants experienced the theory-based behaviour change components embedded within e-coachER. We present the themes in line with our research aims: to report the perceived usefulness of e-coachER and to explore the possible mechanisms of change, specifically issues related to competence, autonomy and relatedness that were identified. The themes were: ‘catalyst for change’, ‘goals and aspirations’, ‘support’ and ‘engagement’.

## Catalyst for change

Most participants had already expressed an intention to make changes to their health, recognised the value of increasing physical activity and were eager to undertake an exercise programme, such as that offered by an ERS. However, e-coachER provided an additional source of motivation. One participant alluded to being motivated to change by e-coachER because it triggered conscientiousness to adhere and a sense of accountability, which was accentuated by a sense of being prompted and monitored:

I think if you embark on something you feel a little bit of a conscience to do it you know you’ve said yes you’ll do it and they send you the stuff in the post so but all these things act as sort of like somebody giving you poke in the arm because at the end of the day it’s in your head and you know you’ve got to go and do it and you might mutter and mumble but because there’s somebody at the other end monitoring what you do it’s going to sort of be a little bit a stick isn’t it than a prompt to you to move forwards. So I think that side of things that’s a good motivator. (P17)

Keen to move forward with their intention to increase physical activity, other participants described how e-coachER was able to provide a further ‘incentive’ (P02) to put into practice their inclination to increase their physical activity. Some participants viewed e-coachER as a catalyst or ‘trigger’ to undertake activities to increase their physical activity:

I think that’s where I would shout the praises of this e-coachER programme is, it triggers you. (P03)

The arrival of the e-coachER welcome pack was also a stimulus for change: the pack was a consequence of joining the ERS, and the ERS referral came from a credible source, their doctor:

it did start me thinking and actually being given the e-coachER pack from my doctor—it was almost like the motivation that I needed to get going. (P13)

In addition, the information provided in the e-coachER steps further augmented the participants’ inclination to change by providing a clear meaningful rationale for engaging in physical activity, thereby fostering a sense of autonomy:

learning that it’s important to build your muscles up to strengthen your bones as well has made me more determined. (P06a)

### Goals and aspirations

A major part of the interviews was focused on goal setting and self-monitoring activities concerning the pedometer and recording strips. Some participants concentrated on establishing Specific, Measurable, Achievable, Relevant and Time-Bound (SMART) goals, while others opted for ‘realistic goals’. Among them, some centred their goals around daily step counts, spanning from 2000 to 10 000, while others focused on particular activities. Participants viewed the goal-setting challenge and subsequent action planning as important features of e-coachER, and they valued the opportunities for self-monitoring:

I know the value of goal setting in life in general. (P07)

The specific goal-setting features of e-coachER, including the provision of a pedometer, were described as something positive for people to focus on. The emphasis on building confidence and competence through e-coachER’s gradual steps also supported participants who already appeared inclined to make changes to their physical activity:

I suppose it did make me think right you don’t have to do everything all in one go you can do a bit at a time. (P14a)

I only set goals that I wanted to set they didn’t force me into setting goals you know you could set your own goals like I just set swimming and walking you know setting your own goals I found quite, if I had been forced to set goals I don’t think I would have, you know, do certain goals, but setting my own goals helped. (P20)

Participants found that the pedometer provided them with a prompt, increasing the salience of their goal to be more physically active while also serving as a reminder of their motivation for doing it:

It [pedometer] tells me “right I’ve got something to do tomorrow so I need to rest up because I’m going to head out”, and you start to make plans. It gets you motivated and all of a sudden you think “well do you know what, yes I am pretty overweight and […] having lots of pains all over my body but tomorrow I am going to do something positive and attempt to right the fact that”—and that helps. That really does help—that’s pure motivation. (P05)

For some, the role of self-monitoring and goal setting invoked feelings of both controlled and autonomous motivation. For example, one participant suggested that their desire to meet their goal was to avoid shame, suggesting more controlled regulation:

It’s just it’s another tool that you can use to, you know, either monitor or encourage or, you know, everyone works differently I suppose and I didn’t, what I needed was something concrete goal-wise to do and to achieve and like I say you know once I’d set that goal then and if I don’t reach it well shame on me kind of thing. (P16)

The importance of autonomy in goal setting was further highlighted, as participants described the need to be in control of setting their own goals:

I feel like if I don’t set myself these goals then I won’t. I would just get to the point where I can feel like you know well I’ve done yesterday so if I do another five more today then it’s not so bad so I’m trying to encourage me to do more than that you know what I mean because setting these goals means a lot to me because that’s what I want to do at the end of the day and if I don’t do them at some point I will I feel bad about it because that’s the point. (P030118)

Achieving or exceeding step goals combined with a desire ‘not to fail’ to meet these goals was also motivating. Participants therefore set their goals to avoid feelings of failure and/or guilt:

Yeah about like I say like you keep them realistic don’t you so you don’t you don’t set yourself up to fail. (P04)

The e-coachER intervention was designed to encourage people to self-monitor their steps and explore achievable step goals. However, to avoid the feeling of failure, some participants wanted to explore this outside of the e-coachER programme first (eg, using pen and paper) rather than officially logging it in the web-based system:

I haven’t as yet put anything down on e-coachER simply because I put down on a piece of paper what I hope to do on a given week and failed miserably to do them so I thought I didn’t want to start to put them down erm until I knew that they were reasonable goals that could be achieved not pipe dreams that have got no chance of being achieved. (P01)

This cautious approach to using the system for logging goals was echoed in the frustrations expressed by other participants, who recognised the need for change but faced numerous difficulties that prevented them from achieving their goals:

Just frustrating to be honest because I can’t fit in everything I want to. The other issue I have is also I am quite sleep deprived as well so that and that’s a vicious cycle of I am having the problem not getting enough good quality sleep and then wanting to eat the wrong things and sit on the couch and then when I have got a minute or an hour or whatever I am loading the washing machine up and cooking for my little girl and doing what you know so it’s but it did make me think for actually if I am going to be a good mum I need to look after myself as well. (P22)

### Support

The e-coachER intervention was designed to highlight the benefit of support, linking participants to others to enhance relatedness. Participants recognised and referred to a ubiquitous and intrinsic need for relatedness, including social praise:

If you’re a small child you’re encouraged to sort of walk aren’t you and everybody applauds you and claps and as a child you feel sort of pretty spectacular because you’ve done something and found your feet and everybody needs encouragement. (P17a)

Participants also discussed how e-coachER encouraged them to seek support in less direct ways. For example, e-coachER enhanced their confidence to talk about their involvement with ERS with others, creating opportunities for others to offer support:

I think probably rather than me seeking that support I think it’s given me the confidence to talk about it, which has then […] helped me seek the support in a roundabout way by talking about it and then them saying “yes we’ll support you in this”. (P24)

Participants recognised that using social support to create a commitment, perhaps through an informal verbal agreement with others, also helped participants maintain their motivation to achieve their goals:

It’s hard yeah hard to motivate yourself and it’s also—I think goals are good as well, that you can tell other people what your goal is and that pushes you to get on with it because other people know what you’re aiming for. (P07)

Similarly, some participants felt that the e-coachER package was instrumental in ameliorating any feelings of guilt they experienced when they felt they had not adequately increased their physical activity. For example, in the latter steps of the intervention, there was a section dealing with ‘slips, trips and falls’, which indicated that failing to meet set goals is normal and something that should be expected. This reassurance was helpful for some:

Being someone who suffers from depression I understand about triggers and setbacks […] But you know this part of the system is explaining to you that it’s ok, don’t worry about it, there are always going to be times in your life where you’re not going to be able to do this and you shouldn’t beat yourself up too badly about it. (P01)

The e-coachER intervention encourages participants to actively seek support; one participant described that e-coachER reminded them to nurture existing support networks:

again I had excellent support already. From erm my GP and my local psychiatry team but they were very supportive about accessing the exercise on prescription and using well I think it was my GP that put me forward through e-coachER. He’s really happy for me to be involved in this so yeah everybody and even my friends who now live in America they’ve all been really, really supportive.

Interviewer: Right was e-coachER helpful in accessing that support for yourself or was…?

Erm not that helpful I think it just reminded me to talk about it. (P04a)

However, the technology of e-coachER could not replace contact with individuals:

As much as we are embracing the technology of life and all the rest of it there is nothing more valuable than a voice and like talking to you. (P21)

In contrast, some participants felt that e-coachER provided a valuable source of support, especially if they lacked social support from others:

whether it’s because I live by myself I don’t know, but I should imagine the people who haven’t got e-coachER for the support, it must be quite hard really [….] I think you know if you live by yourself it’s the only support you get really. (P06)

### Perceived engagement with the e-coachER programme

Many valued e-coachER, considering it to be widely accessible and easy to engage with. People engaged with e-coachER differently, with some stating that they used it regularly (ie, positively engaging with step 3 (step counting and or using the pedometer)) and others finding that just starting e-coachER was enough to motivate them to increase their physical activity:

… it’s [the e-coachER website] given me support as well […]…again, it’s changed my attitude to exercising because as I say I wasn’t very keen to do it, but now I’ve done it I’m enjoying it and I have enjoyed it from the start because—it’s just I’ve seen the results basically and that’s really given me a boost … (P02)

Although e-coachER was initially regarded as easy to engage with, several competing priorities (eg, childcare) affected some participants’ engagement over time:

My position is that I am a single parent so I was finding it quite tough to be able to exercise juggle looking after my daughter and doing a job as well. […] I find it quite frustrating because even though I wanted to do more my time was very much taken up by other things. So yeah it was a little bit frustrating because I was getting quite good tips but I was actually unable to do a lot of it. (P22)

While e-coachER was designed to be simple to increase its accessibility and usability, many found e-coachER to be too simplistic, undermining its credibility:

I suppose you’ve got to make it simple enough so everybody can use it but to me it was too simplified. (P07)

Participants reported that they valued the pedometer despite sometimes experiencing difficulties with the device. For example, some found that it did not work properly, inaccurately recorded steps or was difficult to open or wear. Some participants’ enthusiasm for the pedometer waned with prolonged usage:

Initially I had it on for a month and I was really good tracking it and then I have to be honest, I have it on just now, it kind of petered out a little bit but to start with I you know when you get something and it’s new and it’s you’ve a lot of enthusiasm and you put it on and you track it religiously and it did make me actually when I was looking at it going oh I’ve done 8,000 steps today or I’ll take the dog around the block it did make me do more so that was really good. (P22)

Pedometers were offered with e-coachER as they provided a way of encouraging self-monitoring of steps, a relatively simple physical activity metric for patients to interpret. However, the availability of other wearable devices (eg, Fitbits or other similar devices) may have undermined the pedometer by offering additional features, which were considered of more value to the participants (eg, calorie counting, linking with other apps, mobile phones). In addition, some participants alluded to how they were increasing their sense of autonomy about physical activity; they did not want it to be just a matter of step counts:

I didn’t want to use the pedometer because I didn’t want to just make it about steps it appeared to me that with the pedometer it just made it about how many steps. (P25)

## Discussion

### Summary of findings

We identified four inter-related themes: ‘catalyst for change’, ‘goals and aspirations’, ‘support’ and ‘engagement’. Most participants were keen to engage with e-coachER, describing it as a useful resource. Participants felt the e-coachER was particularly good at motivating them in the initial period of making changes to physical activity, despite feeling that the components of e-coachER were too simple or that the pedometer could have been better.

Our research revealed that recognising a desire to improve one’s health and receiving a comprehensive welcome package with straightforward registration instructions were pivotal elements determining engagement with e-coachER. These results align with previous studies, underscoring the importance of motivation for health behaviour change, visual attractiveness and transparent registration processes as fundamental influences on the decision to explore internet-based interventions.^29^ The findings of our study also highlight the challenge of achieving sustained behaviour change through a digital support programme. Many participants expressed the importance of competency, autonomy and relatedness for behaviour change. However, some participants exhibited more controlled motivation, such as avoiding shame, indicating that additional support may be necessary. Another study, conducted on a group-based physical activity and behaviour maintenance intervention for older adults found that perceptions of autonomy, competence and relatedness were associated with physical activity with group interactions seen as a key source of motivation.^30^ While e-coachER lacked group interaction, the provision of a clear and meaningful rationale for engaging in physical activity was seen as a motivating factor, empowering participants to take control over their health. These findings align with existing literature that highlights the importance of providing a rationale to support autonomy in behaviour change interventions,^31^ suggesting that by engaging participants in understanding the reasons behind engaging in physical activity, they are more likely to embrace it as a positive and empowering experience.

All but two participants in our sample were attending the ERS for weight loss, low mood or both. Previous qualitative studies highlight how many patients with low mood/depression and obesity report low motivation, lack of confidence and stigma as barriers to physical activity.^32 33^ Our findings build on this research, showing that providing behavioural support may help people with low mood and obesity overcome these barriers, enabling them to gradually incorporate physical activity into their lives in a self-selected and achievable way.

Participants in the present study seemed to value the importance of physical activity for health and well-being when entering the study. The e-coachER programme appeared to further elevate this sense of importance, providing a meaningful rationale for engaging in physical activity. This also mirrored what was found in the e-coachER process evaluation, which found that e-coachER led to increases in MVPA at 12 months via increases in the importance of engaging in physical activity.^20^ Systematic review evidence has also supported the notion that identified regulation (ie, exercising because one values its outcomes and desires to maintain good health) tends to predict initial short-term adoption of physical activity.^34^

A key component of e-coachER in these initial stages was the opportunity for self-monitoring and goal setting. Goals in e-coachER were set by the participants, and in line with the theoretical underpinnings of e-coachER, the pedometer facilitated autonomy (setting personal step count and other physical activity goals) and competence. Many participants described the value of being able to set goals and indicated that this enhanced their sense of autonomy and competence. Identifying and setting realistic, meaningful and achievable goals is a key technique in promoting competence within the framework of SDT.^31^ However, previous literature has found that providing structure (including goal setting) can negatively predict autonomy.^35^ This may reflect the fact that goal setting can be promoted in either an autonomous or a controlling manner. Our findings suggest that participants generally perceived goal setting as being promoted in a more autonomous way. Lambert *et al*^20^ also found that e-coachER led to increases in MVPA at 12 months via changes in the use of action planning and self-monitoring. Previous research has also highlighted the importance of autonomy support at the beginning of ERS. For example, in a secondary analysis of 347 adults about to start an ERS, Rouse *et al*^9^ found that autonomy support for more autonomous regulations leads to more positive intentions to be physically active. Donnachie *et al*^36^ similarly highlighted the role of a pedometer in providing tangible evidence of progress, demonstrating enhanced competence and the device being seen as an ‘ally’ in achieving physical activity goals.

Setting SMART goals seemed to be an important aspect of e-coachER, allowing participants to ‘set the pace’ for increasing their physical activity and feel competent and in control. It is also plausible that e-coachER supported self-efficacy to engage in physical activity. For example, previous research highlights how goal setting and self-monitoring enhance self-efficacy, contributing to a person’s belief and skills in initiating and maintaining behaviour change.^37^ Systematic reviews have also found that mechanisms from social cognitive theory (which include self-efficacy) consistently mediate the effects of interventions on increased physical activity.^38^ It is important to remember that e-coachER targeted competence, which, unlike self-efficacy, emphasises the personal relevance of the goal. However, it is reasonable to assume that if e-coachER increased competence, it also increased self-efficacy, given its broader conceptual focus.^39^

Participants generally made more use of earlier e-coachER steps, with 92% of those registered making it to step 2 (support to get active). However, as evident from the interview and usage data, enthusiasm for the package, including self-monitoring and goal-setting components, waned over time.^11^ This suggests that e-coachER was more valuable in the early stages of changing physical activity; previous research has suggested that goal-setting activities are beneficial even when only used for a brief duration (eg, 1 week) and are not necessarily more effective over longer intervention durations.^40^

Previous research has also found that self-monitoring may have a detrimental effect on maintaining motivation.^41^ For example, in a study exploring peoples’ views on weight loss, many expressed a dislike for calorie-counting apps. However, counting calories may be deemed as more difficult than step counting, which can be done with minimal effort (eg, via a pedometer). In addition, while most participants were enthusiastic about obtaining feedback from the pedometer, some were disappointed by the quality of the pedometer and did not feel confident that the step count was accurate. Whether these problems led to participants disengaging with e-coachER is unclear. However, previous research has suggested that mistrust of monitoring equipment may be detrimental to progress.^42^ The e-coachER programme encouraged participants to experiment with other more sophisticated technologies for monitoring personal activity levels, acknowledging the limitations of the low-cost pedometer provided in the intervention. Technology related to self-monitoring devices is evolving rapidly, driven by smartphones and wearable technology. As self-monitoring is known to be an important behaviour change technique, it is crucial to gain a deeper understanding of how the self-monitoring components of online support packages, such as e-coachER, can be effectively used to facilitate, motivate and augment efforts to increase and maintain physical activity.

Participants’ experiences and engagement with e-coachER were influenced by their personal circumstances and may have also been affected by the reasons for their original ERS referral, their health beliefs and other perceived barriers and facilitators to exercise. Participants had clear and detailed reasons for why it was—or was not—possible or desirable to engage with the e-coachER, whether as a whole or with specific components. Engagement in an intervention aimed at changing a particular health behaviour is a precondition for effectiveness.^25^ Being able to use e-coachER flexibly to suit the individual’s unique circumstances could therefore be an important aspect of e-coachER, facilitating autonomy and addressing relatedness and competence needs.^15^ For this reason, greater ‘depth’ to the package, or enhanced functionality as people move through the steps, may be required.

Social support was highlighted in e-coachER as an important component underpinning behaviour change. Many participants had existing access to social support for increasing physical activity and recognised its value. However, for those who did not, e-coachER appeared to help them seek opportunities to enhance relatedness. This finding partly supports the quantitative findings, which indicated that e-coachER led to increased availability of support compared with ERS alone. However, the availability of support did not appear to mediate the effect of e-coachER on increases in MVPA at 12 months.^20^ This lack of change could be attributed to variations in support. Sometimes, it was the contact with ERS or e-coachER staff that provided an important source of support within the e-coachER package, rather than the online support components. The variation in the level of engagement with all e-coachER components is in line with previous research, which has highlighted that user engagement is influenced by a variety of socio-contextual factors, such as family members and the wider cultural environment.^25^

### Strengths and limitations

A particular strength of this qualitative research is that a relatively large number of participants were recruited for the interviews. Participants were keen to provide constructive feedback regarding the contents of the intervention. However, several limitations should also be noted. First, it is unclear to what extent the interviews may have affected the participants’ interaction with the e-coachER support tool. It is also unclear whether the participants’ appreciation of being in the ‘additional interview’ intervention arm affected their responses to the interview questions; some participants were apologetic about being critical of the intervention components and participants may have felt obliged to modify their criticism and instead give more socially desirable feedback on e-coachER. Second, we did not interview any control group participants, nor were we able to interview them towards the end of the main trial, around 12 months postenrolment. This limited our ability to explore the experiences of intervention group participants concerning their long-term quantitative outcomes. Third, as only 27% of participants engaged in more than one interview, we were unable to conduct sufficient follow-up interviews to warrant longitudinal analysis regarding changes in experiences over time. We, therefore, combined data analysis across different time points without considering within-person changes between the different interview time points and focused on the specific steps in the intervention that participants were discussing. Fourth, our study took place within the context of UK-based ERS, potentially making it less generalisable to another context without ERS. However, the e-coachER intervention was designed to encourage physical activity, both within and outside the ERS context, providing flexibility to accommodate differences in the ERS involved in the study. For example, unlike Plymouth and Birmingham, Glasgow ERS supported participants with behaviour change counselling and signposting to different physical activity options, whereas Plymouth and Birmingham offered a more structured and prescriptive approach. Fifth, we refrained from gathering extra demographic and personal details that could have enhanced our understanding of participants’ engagement with the e-coachER intervention. Instead, we opted for a pragmatic approach, concentrating on essential variables like age, gender, health conditions and computer proficiency and accessibility. This decision aimed to alleviate the burden on participants who were already involved in the primary trial data collection.

## Conclusions

For many participants, e-coachER—particularly the pedometer and the associated goal-setting activities—was motivating in the early stages of initiating behaviour change. Our findings highlight the challenges of achieving sustained physical activity changes, suggesting that shifts from extrinsic to intrinsic motivation may be crucial. Further research is required to understand how an online support package such as e-coachER could be developed to provide long-term support for maintaining behaviour change.

## supplementary material

10.1136/bmjopen-2023-080472 online supplemental file 1

10.1136/bmjopen-2023-080472 online supplemental file 2

## Data Availability

Data are available on reasonable request.
