# Supplementary figures and images for: Mechanisms of impact of web-based support and self-monitoring to augment and maintain physical activity levels: a qualitative study exploring participants’ interactions with the e-coachER, a web-based support programme for people attending exercise referral schemes
Source: BMJ Open. 2024 Oct 29;14(10):e080472. doi: 10.1136/bmjopen-2023-080472 (PMC11529766; doi:10.1136/bmjopen-2023-080472)

## Appendix 1

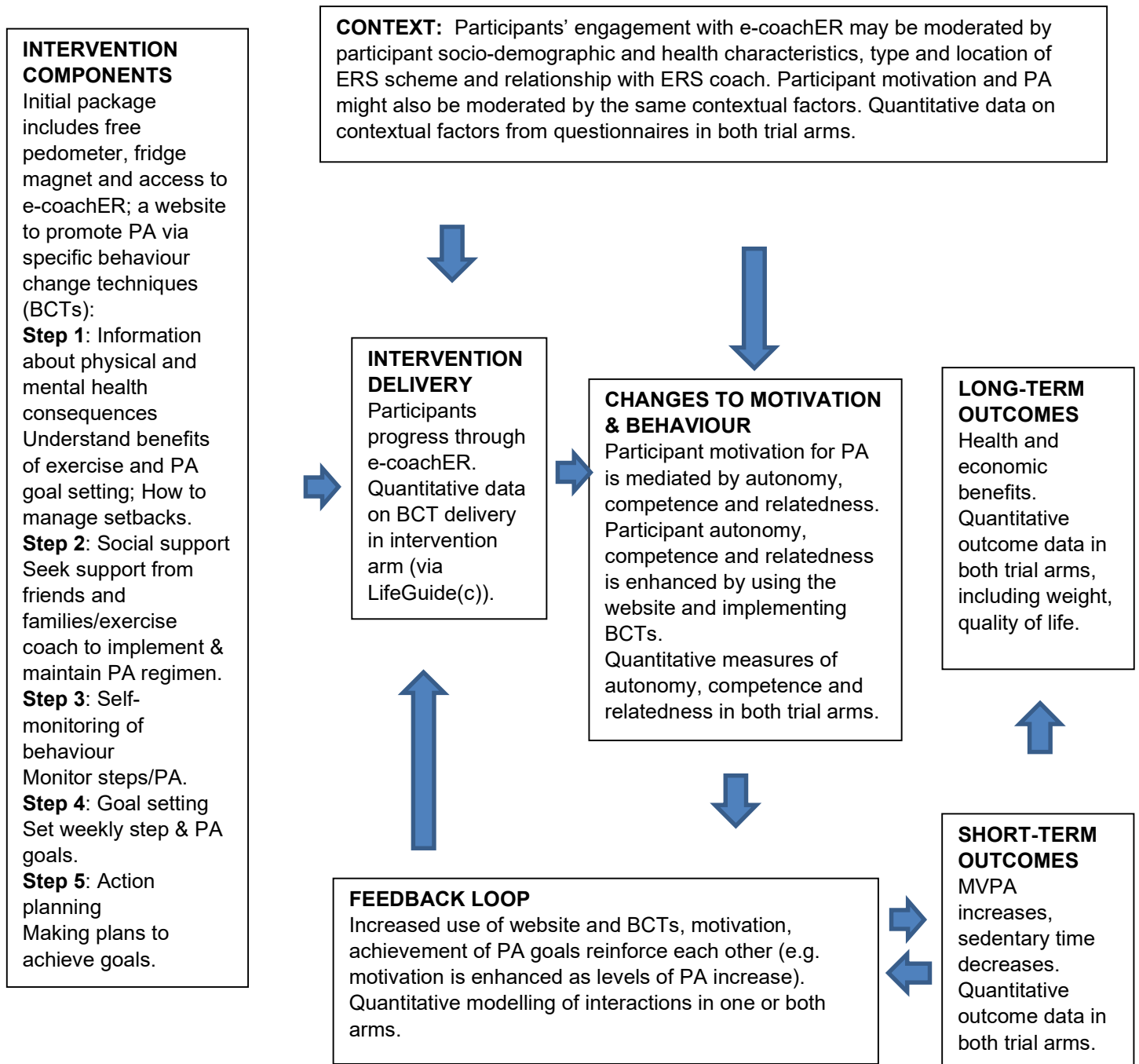

Supplement: online supplemental file 1 [file bmjopen-14-10-s001.pdf]
