# Supplementary material for: Mechanisms of impact of web-based support and self-monitoring to augment and maintain physical activity levels: a qualitative study exploring participants’ interactions with the e-coachER, a web-based support programme for people attending exercise referral schemes
Source: BMJ Open. 2024 Oct 29;14(10):e080472. doi: 10.1136/bmjopen-2023-080472 (PMC11529766; doi:10.1136/bmjopen-2023-080472)
Supplement: online supplemental file 2 [file bmjopen-14-10-s002.pdf]

Appendix 2: Participant telephone interview topic guide

Preamble script

Thank you very much for agreeing to participate in this phone interview in order to help us understand what it has been like to be part of the e-coachER study, we really appreciate your time.

Just to recap on the information we sent you, the purpose of this interview is to understand your experience of e-coachER and any impact it may have had so that we can learn for future development. Please say anything you wish, we want to hear all types of feedback and are keen to hear your views on how things might be done differently to improve the study.

The interview will take around 45 minutes and will be audio-taped to ensure that we do not miss anything. All information you provide will be anonymised; if we use any quotes from you we will not give your name but use a false name.

Before we begin do you have any questions about doing the interview?

Are you therefore willing to give consent to do this interview . . . thank you.

When we are ready to start I will switch the recorder on, say your name and the date; is that OK?

OK so the recorder is now going on . . .

Take verbal consent

Background

- Can you begin by telling me about why you were prescribed the exercise referral scheme?
- How did you hear about e-coachER?
- Have you been referred to an exercise centre? Which one? Has a programme been devised for you?
- You should have received a welcome pack in the post – what did you think of the welcome pack?
    - How did you find the user guide?
    - Is there anything you'd recommend we changed about the user guide?
- Did register on e-coachER website? If no:
    - It is not a problem that you decided not to visit the website; but we are keen to find out your reasons so we can change things for the better in the future . . . so please will you tell me a little bit more about why you did not go to the website? Were there any technical barriers/problems . . . was something else putting you off . . .
- In general what did you think of the website? (Prompt – what were your first impressions? We will go through it in more detail in a minute)
    - So did you register? How was it to do this? . . . is there anything that could be changed to help make registering easier?
    - There was also an e-coachER facilitator to help you with the technology . . . did you make use of this? . . . in what ways was this helpful? Tell me more about the help you received . . . or would have liked to have been given?
- In Step 1 there was a quiz
    - Do you remember doing this?
    - What did you think about the quiz?

    - What were its key messages about the benefits of activity for someone with your condition?
- In Step 2 you were encouraged to find support to get physically active. Can you tell me more about how you used this part of e-coachER?
    - Did you involve family or friends?
- In Step 2 you were also introduced to the 'Links' pages on the website which gives information about local exercise referral schemes and other local support for becoming physically active. These pages also provided general information about becoming physically active.
    - Did you use the links?
    - What did you think about the information provided?
    - Were the links page useful?
    - What links were most helpful?
    - How did you use this support?
- In Step 3 you were asked to use the pedometer to count your steps. This is the little device you wear on your belt.
    - How did you get on with using this?
    - In what ways was it useful for you to use the pedometer?
    - In what ways was the pedometer difficult to use?
    - Is there anything else you'd like to say about the pedometer, anything we should do differently?
- In Step 4 you were asked to set step count goals.
    - What did you understand about the purpose/usefulness of setting these goals?
    - Tell me more about your goal-setting:
    - Was it easy to set step count goals that were . . . specific . . . achievable . . . realistic?
    - In what ways was the pedometer helpful for achieving your step count goals?
    - . . . it was useful (for measuring/seeing progress)?
    - . . . it was not helpful because?
- In the welcome pack there was a fridge magnet with tear off strips to record your steps.
    - Did you use these?
    - Did you put them up on your fridge (or elsewhere)?
    - How have you used these strips to record your steps?
    - How have you found these strips useful or not useful?
- In Step 5 you were asked to make some physical activity plans.
    - Did you use this step to make plans for moderate physical activity?
    - In what ways was it easy or hard to set weekly goals?
    - How did you find the advice about setting SMART goals helpful or unhelpful?
    - In what ways was it easy or hard to keep to a weekly goal?
    - In what ways was it easy or hard to review your weekly goals?
    - In what ways was reviewing your step goals helpful or unhelpful?
- In this step there was some advice on other opportunities to be physically active, for example, travel, leisure time, household chores.
    - Did you find this advice helpful or useful?
    - What did you think about the progress graph? . . .
    - What did you think about the personalised feedback? . . . was the praise . . . encouragement helpful?
    - What was it like not to achieve your goals?

    - To what extent have you used e-coachER to set yourself new step goals each week?
- In Step 6 you were asked about finding ways to help you achieve your physical activity plans. Dealing with the influences in your environment on your physical activity.
    - Did you use this part of the website? How helpful did you find the advice?
    - Please tell me a little more about what you did?
    - Did you make any changes, for example to your daily routine in order to meet your goals?
    - What did you find most motivating?
    - Did you make use of the motivational messages/text/e-mails?
- In Step 7 you were asked to identify any barriers or obstacles to carrying on with your physical activity plans. . . . how did you get on with this task?
    - Were you able to identify any causes of stopping your activity programme? (e.g. something to do with your health condition . . . holidays . . . sickness . . . change at work/caring, etc.)
    - In what way have you found it easy – or not – to challenge negative thoughts about not doing your planned physical activity?
    - Do you feel you have learned how to plan and avoid lapses in physical activity in the future?
- I would like to ask you some more general questions about e-coachER.
    - How relevant was it for you?
    - Overall, how did it help you to set and manage your own goals to increase your physical activity?
    - To what extent did it provide you with new information?
    - How well were you able to engage with e-coachER?
    - How easy was e-coachER to navigate? (e.g. layout of 'steps to health'/main menu, goals)
    - How was the general tone of the website? (Was the language appropriate? Was it supportive? Were the success stories relevant/helpful?)
    - What did you think about the structure/look of the website? (e.g. font size, colour, length of sessions, ability to unlock sessions after set time period).
    - When did you use e-coachER – where were you?/what were you doing?
    - What was the most useful aspect of the e-coachER support package?
    - Is there anything else that we have not talked about that you would like to discuss about e-coachER?
    - Did using e-coachER support you in the ERS?
    - Was e-coachER useful on its own?

Thank participant for their time, etc.
