# Supplementary material for: Mechanisms of impact of web-based support and self-monitoring to augment and maintain physical activity levels: a qualitative study exploring participants’ interactions with the e-coachER, a web-based support programme for people attending exercise referral schemes
Source: BMJ Open. 2024 Oct 29;14(10):e080472. doi: 10.1136/bmjopen-2023-080472 (PMC11529766; doi:10.1136/bmjopen-2023-080472)
Supplement: Supplementary file 3 [file bmjopen-2023-080472.reviewer_comments.pdf]

PEER REVIEW HISTORY

BMJ Open publishes all reviews undertaken for accepted manuscripts. Reviewers are asked to complete a checklist review form and are provided with free text boxes to elaborate on their assessment. These free text comments are reproduced below.

ARTICLE DETAILS

Title (Provisional)

Mechanisms of impact of web-based support and self-monitoring to augment and maintain physical activity levels: A qualitative study exploring participants' interactions with the e-coachER web-based support programme for people attending exercise referral schemes.

Authors

Lambert, Jeffrey D.; Dean, Sarah Gerard; Terry, Rohini H; Charles, Nigel; Greaves, Colin; Campbell, John L; Taylor, Adrian

---

VERSION 1 - REVIEW

**Reviewer**	**1**
**Name**	**O'Brien, Kelly**
**Affiliation**	**University of Toronto, Department of Physical Therapy**
**Date**	**25-Mar-2024**
**COI**	**None.**

The aim of this study was to describe the experiences with the of e-coachER, a digital intervention to support uptake and sustainability of physical activity for people attending exercise referral schemes in the UK. This manuscript addresses and important intervention aimed at enhancing engagement in physical activity in populations of persons with chronic conditions. The manuscript describes a qualitative descriptive sub-study embedded within a larger RCT evaluating the effect of the e-coachER that has potential for applicability across chronic conditions. Below are a number of considerations for revision.

1) Clarity and consistency of the study purpose – the study purpose is unclear and inconsistently stated across different sections in the manuscript, as indicated in the title (experiences of and engaging with…), which differs from the study purpose in the abstract (perceptions of utility and functionality of e-coachER and processes of change). The introduction refers to fidelity, quality of implementation and causal mechanisms and contextual factors associated with outcomes ecoachER – these are all different concepts that require different mechanisms of inquiry and analytical approach.

o There should also be clear study purpose or aim indicated at the end of the introduction that clearly indicates the purpose with the concept of interest, context, population, and timeframe.

2) Specificity of the target population – the target population for the study is unclear and not indicated in the purpose statement in the abstract or introduction. Authors mention 'five clinical conditions' on page 8, but unclear what the conditions are. It is not until the characteristics of the participants that 'health condition' is provided – however it is not clear what the target population is. Weight loss is not a health condition. How is 'low mood' defined? Was the target population persons with obesity and/or mood conditions? Need clear definitions and eligibility of the target population, and inclusion criteria provided. Furthermore, the background / literature review of the manuscript should include a more in-depth review of the literature on physical activity among persons with obesity and mood conditions, and previous literature on digital PA interventions with these populations.

3) Timeframe of the intervention. This should be specified in the purpose statement and the study design. Furthermore, it is unclear at what timepoints the interviews were conducted over the course of the intervention. This is important for readers to understand the nature of the data collection.

4) E-coachER – the e-coachER lacks description and it is also unclear what level of physical activity it is meant to foster – is the aim for the device to help persons achieve greater than or equal to 150 min of moderate to vigorous exercise weekly?. In the results the reader learns more about the device and that it also includes a pedometer. Unclear whether this is an App? A website? A wearable device? In some areas it is referred to as 'programme' – there seems to be goal setting associated with the device but unclear how this process occurs. As well as support? Suggest authors include much more detail in the introduction / background on what the e-coachER is, how it was developed, what components it includes, how it is meant to be used by the participants.

5) E-coachER usage – in order for readers to interpret the qualitative data, it would be helpful to include some general characteristics on uptake and usage of the intervention.

o What does 'ER' stand for in e-coachER?

6) Longitudinal Study Design – This is a qualitative descriptive longitudinal study involving a series of interviews. It is unclear the timeframe in which the interviews occurred across the course of the study, and unclear how the analysis was performed with the transcripts over time. More detail on how the data analyses were analyzed for changes in the experiences over time is needed.

7) Characteristics of Participants Table 1 – Site 1, 2 and 3 in Table 1 is not comprehensible, suggest the sites are more clearly defined / described – this is important so that readers can determine and interpret the transferability of findings to other contexts.

o Request additional demographic and personal characteristics of participants that are important as they influence and interact with engagement in exercise; such as employment status, income, social situation and living status, highest level of education, ethnicity, etc.

o Results on page 18 refer to some participants ‘using it regularly’ – but unclear wat this means. More detail on the usage of the device would be beneficial when describing ‘engagement’.

8) Results – a strength of the study is the use of a logic model to inform the study design and analysis, however it is unclear how the 4 themes were derived from the logical model of competence, relatedness and autonomy and how this influences engagement in physical activity. Perhaps a figure of the logic model / conceptual framework would be helpful for the reader to outline how the logic model informed the inquiry and the resulting themes.

o For the goal setting section, it would be helpful to have a better understanding of the type of goals that were set by the participants in order to interpret how the device facilitated the ability to set goals.

o Unclear how results relate to changes over time (within the context of the longitudinal study design).

9) Discussion: How do the findings relate to the literature of self-efficacy of physical activity and exercise.

o More discussion of the findings specific to the target population in this study is warranted – obesity and mood conditions?

o More in-depth review of the literature on the implementation of the device in relation to other digital health interventions with the aim to enhance physical activity would be helpful.

10) Limitations: Authors should discuss the transferability of the findings to other health care contexts, for example how might this work be appliable to other contexts that do not have ERSs? Other health conditions? This seems specific to the UK context, so unclear how and whether the e-coach-ER has a role in other health care contexts.

11) Abstract – more clarity and consistency of the stated study purpose. Design is a longitudinal qualitative study – need to indicate the timeframe and details of when the interviews were conducted over time, and how the data were specifically analyzed longitudinally. E-coachER should be defined and described.

VERSION 1 - AUTHOR RESPONSE

Dr Edward Sucksmith

Executive Editor

BMJ Open

Subject: Point-by-point response to reviewers

Submission ID: bmjopen-2023-080472

Title: On-line support and self-monitoring to augment and maintain physical activity levels: A qualitative study exploring participants' experience of, and engagement with, the e-coachER web-based support for people with long-term health conditions

Dear Dr Sucksmith,

Thank you for considering our manuscript for publication in BMJ Open and for inviting us to resubmit.

We are pleased that you and the reviewer find the article to have merit, and the collective comments and suggestions have been extremely useful to us in our revisions. Changes to the revised manuscript have been highlighted using tracked changes. We would like to thank the reviewer and yourself for your helpful and constructive comments.

Your sincerely,

Dr Jeffrey Lambert

Editorial request

Comment: The Patient and Public Involvement statement should be included as a sub-heading in the methods section. Please ensure that the statement "The e-coachER intervention was developed by researchers at the University of Southampton, Plymouth and Exeter and with Patient and Public Involvement (PPI) input, using the LifeGuide platform (www.lifeguideonline.org (Lloyd et al., 2013; Yardley et al., 2010, 2016)." is placed under the subheading "Patient and Public Involvement".

Response: We have now moved this statement to a separate subheading in the method section. Please see page 9.

Reviewer comments

Comment 1: Clarity and consistency of the study purpose – the study purpose is unclear and inconsistently stated across different sections in the manuscript, as indicated in the title (experiences of and engaging with…), which differs from the study purpose in the abstract (perceptions of utility and functionality of e-coachER and processes of change). The introduction refers to fidelity, quality of implementation and causal mechanisms and contextual factors associated with outcomes e-coachER – these are all different concepts that require different mechanisms of inquiry and analytical approach.

o There should also be clear study purpose or aim indicated at the end of the introduction that clearly indicates the purpose with the concept of interest, context, population, and timeframe.

Response: Thank you for highlighting the concerns regarding the clarity and consistency of our study purpose across the manuscript. The introduction section intended to refer to the wider process elevation of which this qualitative study was part. However, we acknowledge that this is unclear and recognize the importance of ensuring a cohesive presentation. To rectify this, we have consistently applied terminology from the MRC Process evaluation framework across our title, abstract and introduction. We have also refined the aim at the end of the introduction, which states the purpose, concept of interest, context, population and timeframe. Please see page 7. We have also provided the revised aim below.

"This qualitative investigation aimed to explore how e-coachER influenced mechanisms of behaviour change specified in our logic model (see: Taylor et al., 2020). Specifically, how did patients with chronic conditions attending an ERS interact with e-coachER and to what extent did the intended mechanisms of competence, relatedness and autonomy mediate the effect of e-coachER on behaviour change and maintenance of any increases in physical activity?"

Comment 2: Specificity of the target population – the target population for the study is unclear and not indicated in the purpose statement in the abstract or introduction. Authors mention 'five clinical conditions' on page 8, but unclear what the conditions are. It is not until the characteristics of the participants that 'health condition' is provided – however it is not clear what the target population is. Weight loss is not a health condition. How is 'low mood' defined? Was the target population persons with obesity and/or mood conditions? Need clear definitions and eligibility of the target population, and inclusion criteria provided. Furthermore, the background / literature review of the manuscript should include a more in-

depth review of the literature on physical activity among persons with obesity and mood conditions, and previous literature on digital PA interventions with these populations.

Response: The recruitment pathways and inclusion criteria for the main e-coachER study have been reported elsewhere (see: Taylor et al, 2020; 2021). In the UK and elsewhere, ERS have minimal transfer of medical information. As we were unable to depend on a clinical diagnosis from a medical practitioner for all trial participants, we asked participants to self-report their primary reason for a referral from the following: the need to lose weight, diabetes, hypertension, osteoarthritis or a history of depression/low mood. We have added further clarity in the participants section on page 8. We have also added a further review of the literature on physical activity and digital interventions among persons with obesity and mood on page 5.

Comment 3: Timeframe of the intervention. This should be specified in the purpose statement and the study design. Furthermore, it is unclear at what timepoints the interviews were conducted over the course of the intervention. This is important for readers to understand the nature of the data collection.

Response: The e-coachER intervention had 7 weekly steps to health. However, participants had access to the intervention throughout the entire study period. So once all steps had been unlocked, they could still go back and access different modules. We have added this detail in the description of e-coachER in the background on page 6. We have also added further information concerning the time points of the interviews on page 11.

Comment 4: E-coachER – the e-coachER lacks description and it is also unclear what level of physical activity it is meant to foster – is the aim for the device to help persons achieve greater than or equal to 150 min of moderate to vigorous exercise weekly?. In the results the reader learns more about the device and that it also includes a pedometer. Unclear whether this is an App? A website? A wearable device? In some areas it is referred to as 'programme' – there seems to be goal setting associated with the device but unclear how this process occurs. As well as support? Suggest authors include much more detail in the introduction / background on what the e-coachER is, how it was developed, what components it includes, how it is meant to be used by the participants.

Response: e-coachER just aimed to generate a significant increase in MVPA from the participants' baseline level /there was no specific minimum level targeted. e-coachER is a web-based programme. More detailed information has been reported elsewhere (see: Taylor et al, 2020; 2021). We have added more detail about the e-coachER intervention on page 6 in the background section.

Comment 5a: E-coachER usage – in order for readers to interpret the qualitative data, it would be helpful to include some general characteristics on uptake and usage of the intervention.

Response: We have added to following text to page 7 in the introduction:

"Overall, 36% of participants did not log in to e-coachER, and 36% engaged enough to complete at least one goal review, requiring over 4-weeks of interaction. There was a mean of 14.1 logins (SD 16.7, and of those that completed a goal review, there was a mean of 14.4 (SD 13.8) reviews. The mean time spent on e-coachER was 48.4 (SD 41.9) minutes for those who registered and 43.3 (SD 37.3) minutes for those who completed a goal review."

Comment 5b: What does 'ER' stand for in e-coachER?

Response: The ER stand for 'exercise referral'. We have spelt this out on page 6.

Comment 6: Longitudinal Study Design – This is a qualitative descriptive longitudinal study involving a series of interviews. It is unclear the timeframe in which the interviews occurred across the course of the study, and unclear how the analysis was performed with the transcripts over time. More detail on how the data analyses were analysed for changes in the experiences over time is needed.

Response: Thank you for the comment. Due to high attrition from both the main trial and the interview study, we were unable to conduct sufficient follow-up interviews to warrant longitudinal analysis regarding changes in experiences over time. We therefore combined data analysis across different timepoints without considering within-person changes between the different interview timepoints and focussed the analysis on which step in the intervention participants were talking about. However, we appreciate this could be clearer. We have therefore added more detail in the results (page 11) regarding what steps participants reached and whether their data were from a single interview or multiple interviews. We have also added a limitation to the discussion (page 28) and in the strengths a limitations section:

Comment 7a: Characteristics of Participants Table 1 – Site 1, 2 and 3 in Table 1 is not comprehensible, suggest the sites are more clearly defined / described – this is important so that readers can determine and interpret the transferability of findings to other contexts.

Response: We have now added the site names to table 1.

Comment 7b: Request additional demographic and personal characteristics of participants that are important as they influence and interact with engagement in exercise; such as employment status, income, social situation and living status, highest level of education, ethnicity, etc.

Response: Thank you. We agree that gathering extra demographic and personal details could have enhanced our understanding of participants' engagement with the e-coachER intervention. However, we refrained from gathering extra demographic and personal details, concentrating on essential variables like age, gender, health conditions, and computer proficiency and accessibility. This decision was to alleviate the burden on participants who were already completing the primary trial data collection. We have acknowledged this in the limitations section of the discussion.

Comment 7c: Results on page 18 refer to some participants 'using it regularly' – but unclear wat this means. More detail on the usage of the device would be beneficial when describing 'engagement'.

Response: We have addressed this comment in two ways. First, we have changed the text to reflect that this was the participant's perceived engagement with e-coachER instead of objective usage (reported in the main report (Taylor et al., 2020). Second, we have clarified what we meant by the term 'regularly'. See page 20.

Comment 8a: Results – a strength of the study is the use of a logic model to inform the study design and analysis, however it is unclear how the 4 themes were derived from the logical model of competence, relatedness and autonomy and how this influences engagement in physical activity. Perhaps a figure of the logic model / conceptual framework would be helpful for the reader to outline how the logic model informed the inquiry and the resulting themes.

Response: We have added the logic model for e-coachER in Appendix 1. We have also added further clarity in the method as to how the logic model directly informed the design of the topic guide to capture key aspects of intervention delivery, as well as mechanisms of change (e.g., competence, autonomy, and relatedness). See page 10.

Comment 8b: For the goal setting section, it would be helpful to have a better understanding of the type of goals that were set by the participants in order to interpret how the device facilitated the ability to set goals.

Response: We have added further detail around the types of goals participants were setting (page 15). Some participants concentrated on establishing SMART goals, while others opted for "realistic goals." Among them, some centred their goals around daily step counts, spanning from 2000 to 10,000, while others focussed on particular activities.

Comment 8c: Unclear how results relate to changes over time (within the context of the longitudinal study design).

Response: Due to high attrition, we were unable to analyse the data longitudinally and instead amalgamated the analysis across multiple time points. In effect, this adds an aspect of purposive sampling (we collected data at a range of time points after the intervention had been given) but does not amount to a comprehensive longitudinal analysis (which was our original intention). As per our response to comment 6, we have therefore added more detail in the results (page 11) regarding what steps participants reached and whether their data were from a single interview or multiple interviews. We have also added a limitation to the discussion (page 28).

Comment 9a: Discussion: How do the findings relate to the literature of self-efficacy of physical activity and exercise.

Response: Thank you. We provided some discussion around self-efficacy on page 25. However, we appreciate this could have been expanded upon. We have therefore expanded upon this to make a clearer comparison with the findings and self-efficacy of physical activity and exercise. See page 25 and the text below:

Setting SMART (Specific, Measurable, Achievable, Relevant, and Time-Bound) goals seemed to be an important aspect of e-coachER, allowing participants to 'set the pace' for increasing their PA and feel competent and in control. It is also plausible that e-coachER supported self-efficacy to engage in PA. For example, previous research highlights how goal setting and self-monitoring enhances self-efficacy, contributes to a person's belief and skills in initiating and maintaining behaviour change (Greaves et al., 2011). Systematic reviews have also found that mechanisms from social cognitive theory (which include self-efficacy) consistently mediate the effects of interventions on increased PA (Rhodes et al., 2021). It is important to remember that e-coachER targeted competence, which, unlike self-efficacy, emphasises the personal relevance of the goal. However, it is reasonable to assume that, if e-coachER increased competence, it is also increased self-efficacy, given its broader conceptual focus (Rodgers et al., 2013).

Comment 9b: More discussion of the findings specific to the target population in this study is warranted – obesity and mood conditions?

Response: Many thanks, we have added further discussion of the findings related to other qualitative studies in the target population. See page 23 and below text.

All but two participants in our sample were attending the ERS for weight loss, low mood or both. Previous qualitative studies highlight how many patients with low mood/depression and obesity report low motivation, lack of confidence and stigma as barriers to physical activity (Searle et al 2011; Bombak 2015). Our findings build on this research, showing that providing behavioural support may help people with low mood and obesity overcome these

barriers, supporting people to gradually build physical activity into their lives in a self-selected and achievable way.

Comment 9c: More in-depth review of the literature on the implementation of the device in relation to other digital health interventions with the aim to enhance physical activity would be helpful.

Response: We have added some further text in the discussion to relate the implementation of e-coachER to other Internet-based interventions. See page 23 and below:

Our research revealed that recognizing a desire to improve one's health and receiving a comprehensive welcome package with straightforward registration instructions were pivotal elements determining engagement with e-coachER. These results align with previous studies, underscoring the importance of motivation for health behaviour change, visual attractiveness, and transparent registration processes as fundamental influences on the decision to explore internet-based interventions (Brouwer et al., 2009)

Comment 10: Limitations: Authors should discuss the transferability of the findings to other health care contexts, for example how might this work be appliable to other contexts that do not have ERSs? Other health conditions? This seems specific to the UK context, so unclear how and whether the e-coach-ER has a role in other health care contexts.

Response: We have added the following text in the limitations discussing the transferability of the findings to other contexts. See page 28 and the below text:

Fourth, our study took place within the context of UK-based ERS, potentially making it less generalisable to another context without ERS. However, the e-coachER intervention was designed to encourage PA, both within and outside of the ERS context and to accommodate differences in the ERS which took part in the study. For example, unlike Plymouth and Birmingham, Glasgow ERS supported participants with behaviour change counselling and signposting to different PA options, whereas Plymouth and Birmingham were more structured and prescriptive.

Comment 11: Abstract – more clarity and consistency of the stated study purpose. Design is a longitudinal qualitative study – need to indicate the timeframe and details of when the interviews were conducted over time, and how the data were specifically analyzed longitudinally. E-coachER should be defined and described.

Response: Thank you. We have added more clarity and consistency to the study purpose (see response to comment 1). We have also removed ‘multiple interviews’ to avoid misleading the reader that this was a longitudinal study. We have also defined and described the e-coachER intervention.

VERSION 2 - REVIEW

**Reviewer**	**1**
**Name**	**O'Brien, Kelly**
**Affiliation**	**University of Toronto, Department of Physical Therapy**
**Date**	**13-Aug-2024**
**COI**	**I have no competing interests.**

The aim of this study was to describe the how persons with chronic conditions attending an exercise referral scheme interacted with e-coachER (a digital health intervention to support uptake of physical activity involving an online user guide, pedometer, fridge magnet to record physical activity, and weekly health sessions) to boost motivation for and engagement in physical activity. Overall, authors addressed the comments as the revised manuscript includes clarification of the study purpose, target population, description of the e-coachER intervention, and description of how data were analyzed (cross-sectionally rather than longitudinal despite a longitudinal study design with interviews over time). A few minor points for consideration:

1) Description of Characteristics of Participants and their e-coachER usage – the following paragraph should be part of the results (not the background). Suggest this paragraph be moved to the results, after describing the personal characteristics of the participants, to describe the usage of e-CoachER for context.

o Move to results: “Overall, 36% of participants did not log in to e-coachER, and 36% engaged enough to complete at least one goal review, requiring over 4-weeks of interaction. There was a mean of 14.1 logins (SD 16.7, and of those that completed a goal review, there was a mean of 14.4 (SD 13.8) reviews. The mean time spent on e-coachER was 48.4 (SD 41.9) minutes for those who registered and 43.3 (SD 37.3) minutes for those who completed a goal review (Taylor et al., [11,18])”

2) Referencing - there are a few areas in the manuscript Background section where Taylor et al. is indicated (see above)– it might be that these additional references were added at the revision stage. Suggest the referencing is accurate and consistent with BMJ Open journal style throughout.

3) Spelling, Spacing and Grammar

o Procedure section – there is a typo halfway through this paragraph – ‘contectual’ should be contextual.

o Discussion section - For example, previous research highlights how goal setting and self-monitoring enhances self-efficacy,contributes …. [add space between efficacy and contributes]

4) Discussion - Goal Setting – the concept of goal setting is an interesting feature of the intervention and appeared to be a key component to the interviews. Further discussing the e-coachER in relation to the goal setting process and behaviour change in the discussion would be beneficial.

VERSION 2 - AUTHOR RESPONSE

Shona Reeves

Executive Editor

BMJ Open

Subject: Point-by-point response to reviewers

Submission ID: bmjopen-2023-080472.R1

Title: Mechanisms of impact of web-based support and self-monitoring to augment and maintain physical activity levels: A qualitative study exploring participants' interactions with the e-coachER web-based support programme for people attending exercise referral schemes

BMJ Open

Dear Shona Reeves,

Thank you for considering our manuscript for publication in BMJ Open and for inviting us to resubmit.

We are pleased that both you and the reviewer feel we have addressed your comments. Thank you for your additional feedback; we have made the suggested changes to the revised manuscript, which are highlighted using tracked changes. We would like to extend our gratitude to both you and the reviewer for your valuable and constructive comments.

Your sincerely,

Dr Jeffrey Lambert

Reviewer comments

Comment 1: Description of Characteristics of Participants and their e-coachER usage – the following paragraph should be part of the results (not the background). Suggest this paragraph be moved to the results, after describing the personal characteristics of the participants, to describe the usage of e-CoachER for context.

o Move to results: "Overall, 36% of participants did not log in to e-coachER, and 36% engaged enough to complete at least one goal review, requiring over 4-weeks of interaction. There was a mean of 14.1 logins (SD 16.7, and of those that completed a goal review, there was a mean of 14.4 (SD 13.8) reviews. The mean time spent on e-coachER was 48.4 (SD 41.9) minutes for those who registered and 43.3 (SD 37.3) minutes for those who completed a goal review (Taylor et al., [11,18])"

Response: Many thanks for your feedback. We have now moved this paragraph to the Results section, immediately after summarising the participant characteristics.

Comment 2: Referencing - there are a few areas in the manuscript Background section where Taylor et al. is indicated (see above)– it might be that these additional references were added at the revision stage. Suggest the referencing is accurate and consistent with BMJ Open journal style throughout.

Response: We have now removed all instances where the author's name is followed by "et al." in parentheses.

Comment 3: Spelling, Spacing and Grammar

o Procedure section – there is a typo halfway through this paragraph – 'contectual' should be contextual.

o Discussion section - For example, previous research highlights how goal setting and self-monitoring enhances self-efficacy,contributes …. [add space between efficacy and contributes]

Response: Thank you for your feedback. We have corrected these typos and thoroughly checked the manuscript for any remaining errors.

Comment 4: Discussion - Goal Setting – the concept of goal setting is an interesting feature of the intervention and appeared to be a key component to the interviews. Further

discussing the e-coachER in relation to the goal setting process and behaviour change in the discussion would be beneficial.

Response: Many thanks, we have added some additional discussion around goal setting, please see below text.

“Identifying and setting realistic, meaningful, and achievable goals is a key technique in promoting competence within the framework of Self-Determination Theory (SDT) [31]. However, previous literature has found that providing structure (including goal setting) can negatively predict autonomy [35]. This may reflect the fact that goal setting can be promoted in either an autonomous or a controlling manner. Our findings suggest that participants generally perceived goal setting as being promoted in a more autonomous way.”
